# Acetylsalicylic acid potentiates passive systemic anaphylaxis in mice

**DOI:** 10.1186/2045-7022-1-S1-O25

**Published:** 2011-08-12

**Authors:** Maria Nassiri, Magda Babina, Margitta Worm

**Affiliations:** 1 Allergie Centrum-Charite, Klinik für Dermatologie und Allergologie, Campus Charité Mitte, Charité- Universitätsmedizin, Berlin, Germany

## 

Acetylsalicylic acid (ASA) is a nonsteroidal anti-inflammatory drug that can cause mast cell dependent diseases in sensitive individuals. ASA induced asthma is believed to be related to an overproduction of cysteinyl leukotriene C4 (LTC4) secondary to cyclooxygenase inhibition. It remains to be elucidated whether and by which mechanisms ASA may also influence systemic anaphylactic reactions.

In order to clarify if ASA modulates passive systemic anaphylaxis (PSA), Balb/c mice were pre-treated with ASA, sensitized with anti-TNP-BSA intravenously followed by intravenous challenge with TNP-BSA. The temperature profile was assessed for 70 min. Levels of mast cell mediators in the sera (histamine, serotonin, LTC4) were determined by ELISA. Additionally murine bone marrow-derived cultured and peritoneal mast cells were incubated *in vitro* with ASA, loaded with IgE, stimulated with anti-IgE and histamine release was assessed.

ASA aggravated the symptoms of PSA; the maximum temperature drop for ASA pre-treated mice was 5.1 ± 0.4 versus 3.7 ± 0.5 in the control (p = 0.004). In line with exacerbated hypothermia, elevated amounts of mast cell mediators were found in mouse sera. LTC4 was enhanced and most interestingly, increases in the preformed mediators histamine and serotonin were likewise detected. Contrary to these findings the histamine release of mast cells incubated with ASA *in vitro* was reduced.

Together, ASA potentiates PSA probably by enhancing the degranulation of mast cells *in vivo*, thereby increasing the availability of anaphylactic mediators. Since ASA suppresses mast cell activation *in vitro*, the environment surrounding the mast cells dictate most likely changes in the releasability of these effector cells upon ASA treatment. Our findings emphasize the importance of *in vivo* models to study anaphylaxis as only *in vivo* experiments can unravel the complex interplay of different cells and tissue factors.

